# An international review of the frequency of single-bicycle crashes (SBCs) and their relation to bicycle modal share

**DOI:** 10.1136/injuryprev-2013-040964

**Published:** 2014-01-09

**Authors:** Paul Schepers, Niels Agerholm, Emmanuelle Amoros, Rob Benington, Torkel Bjørnskau, Stijn Dhondt, Bas de Geus, Carmen Hagemeister, Becky P Y Loo, Anna Niska

**Affiliations:** 1 Ministry of Infrastructure and the Environment, Delft, The Netherlands; 2 Aalborg University, Aalborg, Denmark; 3 IFSTTAR, Université de Lyon, Lyon, France; 4 Public Health (Bristol), Bristol, UK; 5 TØI Institute of Transport Economics, Oslo, Norway; 6 Vrije Universiteit Brussel, Brussels, Belgium; 7 Technische Universität Dresden, Dresden, Germany; 8 University of Hong Kong, Pokfulam, China; 9 VTI, Swedish National Road and Transport Research Institute, Linköping, Sweden

## Abstract

**Objectives:**

To study cyclists’ share of transport modes (modal share) and single-bicycle crashes (SBCs) in different countries in order to investigate if the proportion of cyclist injuries resulting from SBCs is affected by variation in modal share.

**Methods:**

A literature search identified figures (largely from western countries) on SBC casualties who are fatally injured, hospitalised or treated at an emergency department. Correlation and regression analyses were used to investigate how bicycle modal share is related to SBCs.

**Results:**

On average, 17% of fatal injuries to cyclists are caused by SBCs. Different countries show a range of values between 5% and 30%. Between 60% and 95% of cyclists admitted to hospitals or treated at emergency departments are victims of SBCs. The proportion of all injured cyclists who are injured in SBCs is unrelated to the share of cycling in the modal split. The share of SBC casualties among the total number of road crash casualties increases proportionally less than the increase in bicycle modal share.

**Conclusions:**

While most fatal injuries among cyclists are due to motor vehicle–bicycle crashes, most hospital admissions and emergency department attendances result from SBCs. As found in previous studies of cyclists injured in collisions, this study found that the increase in the number of SBC casualties is proportionally less than the increase in bicycle modal share.

## Introduction

**Figure 1:**
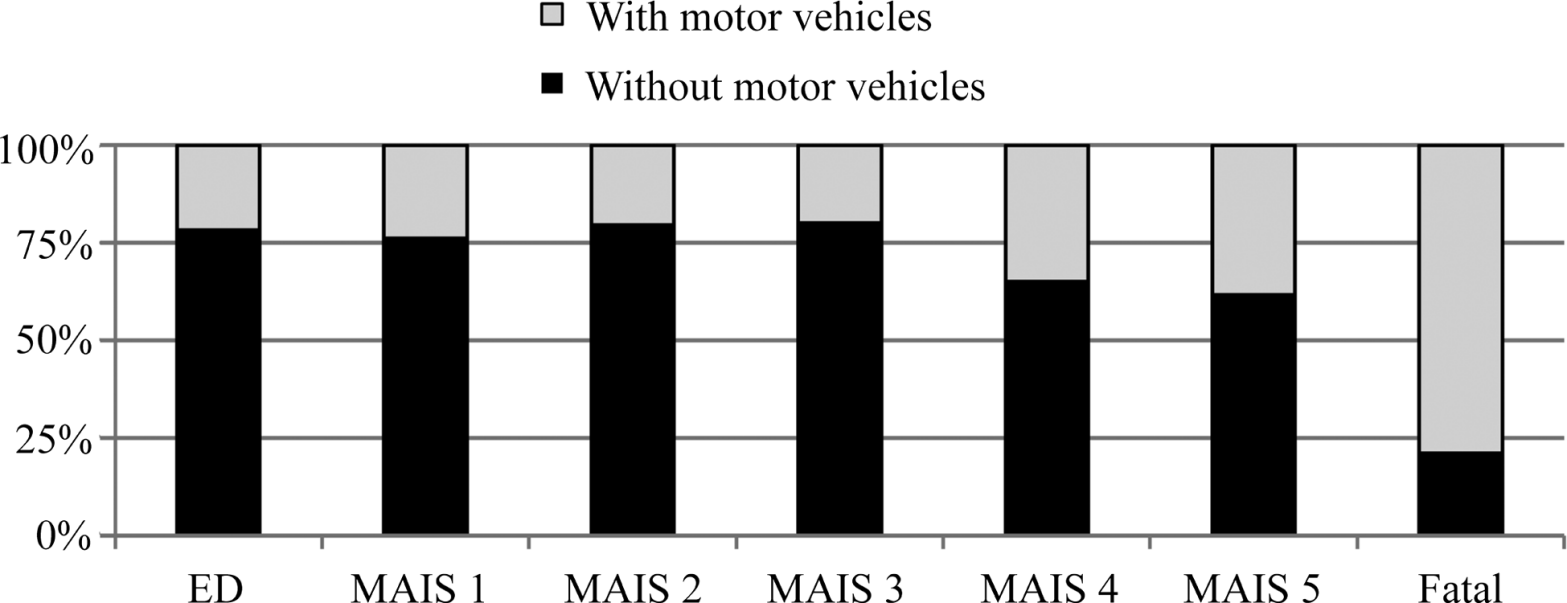
Distribution of injuries incurred by cyclists in crashes with and without motor vehicles in the Netherlands (ED, emergency department).^22–24^

With increasing attention being paid to the promotion of active, low-carbon travel for health, environmental, social and economic benefits, it is important to understand the relationship between bicycle use and incidence of injury. Single-bicycle crashes (SBCs) are a significant^1–3^ and (in some countries) increasing cause of serious transport related injuries.^3–5^ SBCs cause injuries that result in emergency admission to hospital that are coded as ‘non-collision incident’ and ‘collision with fixed and stationary objects’ using the International Classification of Diseases 10. The problem of SBCs has remained hidden for a long time because SBCs are rarely reported in official road crash statistics^6–9^ which do not regularly include hospital data. This may explain why research into SBCs, contributory factors and effects on bicycle use is at an early stage.^10^ It is important to understand SBCs for at least four reasons: (1) SBCs are a significant cause of serious injury, see e.g. figure 1 showing that non-fatal injuries incurred by Dutch cyclists are, regardless of injury severity, mostly due to crashes that do not involve motor vehicles, the large majority being SBCs; (2) SBCs cause direct economic costs through absence from work and from productivity losses;^1^ ^11^ (3) the hazards that lead to SBCs such as poor infrastructure quality may discourage more active travel by bicycle, thereby preventing people from taking advantage of the health benefits of cycling;^12–17^ and (4) there is a moral obligation to understand the risks of activities that are being promoted so that risks can be minimised or removed and potential participants can grant their informed consent to accept the risks that remain. The causes of SBCs are outside the scope of this paper as these are more fully discussed elsewhere.^2^ ^3^ ^9^ ^10^ ^18–21^

The relationship between increasing bicycle usage and bicycle crashes involving collisions has received significant research attention,^25–27^ but, as yet, only one Dutch study by Schepers addressed this issue for SBCs.^28^ Schepers found the increase in SBCs to be proportionally less than the increase in bicycle usage,^28^ mirroring the ‘non-linearity of risk’ found for motor vehicle–bicycle (MV-B) crashes.^25^ ^26^ Most explanations for the non-linearity of risk relate to MV-B crashes. For instance, Jacobsen^25^ suggests that motorists modify their behaviour when they expect or experience people walking and bicycling, which he called ‘safety in numbers’. Different explanations may apply to the risk of SBC: local authorities may improve infrastructure as the amount of cycling increases^22^ and vice versa.^29^ Fewer crashes may occur as cyclists become more experienced and skilled.^28^ To explore whether non-linearity of risk also applies to SBCs, we examined the frequency of SBCs in a sample of countries with varying amounts of cycling.

Using a literature search, we examined (1) the frequency of SBCs resulting in fatal injuries; (2) the frequency of SBCs resulting in severe non-fatal injuries (here defined as casualties who are admitted to the hospital (hospitalised) or treated at an emergency department (ED)); (3) how bicycle modal share is associated with the proportion of cycling injuries occurring in SBCs; and (4) the hypothesis that an increase in bicycle modal share is related to a less than proportional increase in the share of SBCs among all road crash victims. We aimed to include data from countries with varying bicycle modal shares to allow for hypothesis testing. We did not restrict the study to specific countries, but most published research is from Western countries.

## Methods

### Data sources

We searched for SBC and modal share figures from countries with varying bicycle modal shares. In this study, SBCs are defined as falls and obstacle collisions (all crash types in which only the cyclist is involved). Other typical bicycle crash types, not included in this study, are collisions with motor vehicles, cyclists and pedestrians. We used Google Scholar, SafetyLit and Scopus to search for studies about SBCs with ‘single-bicycle crash’ and ‘bicycle-only crash’ as search terms. We contacted research organisations for grey literature and included this alongside published studies. In some cases, we also contacted authors of the publications for additional information. We also searched for literature in which bicycle modal shares are reported to allow for hypothesis testing. The data sources are further described for fatal injuries, hospitalised injuries and injuries treated at an ED. This also includes data on modal share that are used to study the relationship with SBCs.

#### Fatalities

SBCs are rarely reported by the police, but crashes with a higher injury severity, especially fatal crashes, are associated with an increased reporting and recording rate.^7^ ^8^ ^30^ Therefore, we used existing reviews by ETSC^31^ and IRTAD^32^ which included police reported data on fatalities in European countries. Other data sources for fatalities in SBCs are not sufficiently available as yet. We restricted analyses on fatalities to Europe because a study by Gallup^33^ identified modal share in European countries using the same method for each country. Per country 1000 people answered the following question: ‘What is the main mode of transport that you use for your daily activities?’^33^

#### Hospitalised injuries

The search identified 13 studies that included figures for hospitalised casualties from hospital records or questionnaires filled out by hospitalised victims. Studies focused on a smaller area such as a city were excluded in case we found a study for a whole county. Also, if there were two sources for the same area, we included the most recent one only. For purposes of comparability, the categories ‘unknown’ or ‘unspecified’ (in the studies used for this investigation) are excluded from the estimated proportions of SBC victims. Some studies only reported the share of injuries due to bicycle crashes with no motor vehicles, which also includes bicycle–bicycle and bicycle–pedestrian crashes. Based on other studies, it was estimated that 90% were a victim of a SBC.^18–20^ ^23^ ^34^ For seven out of 13 study areas, we could use the study by Gallup to determine bicycle modal share. For five non-European countries, modal share data were obtained from other sources (such as Pucher and Buehler^35^ for Canada, USA and Australia) which are based on National Travel Surveys (NTS). Bicycle modal share in such studies is determined using travel diaries. The data from Gallup and NTSs may vary, which is further discussed in the Analyses section.

#### Injuries treated at an ED

The search identified 13 studies including figures for casualties treated at an ED. The same protocol as for hospital admissions (see Hospitalised injuries section) was followed for selection of studies and data processing. As we could not find data on bicycle modal share for a sufficiently large number of study areas, hypothesis testing is restricted to fatal and hospitalised injuries and excludes ED treatments.

### Analyses

A Pearson correlation is calculated between the share of SBCs and bicycle modal share to examine this relationship. However, the bicycle modal share measure used to correlate with hospitalised casualties contains data from both the Gallup study and from NTS studies. To control for possible differences we have created a control variable that equals zero for Gallup based measures and one for NTS based, that is, a dummy variable. Linear regression analysis is conducted on the share of hospitalised SBC casualties in the total number of hospitalised casualties with bicycle modal share and the control variable for bicycle modal share measurement method as independent variables.

To investigate whether an increase in bicycle modal share is related to a less than proportional increase in the share of SBCs among all road crash victims, trend lines of the form SSBCT=α BMS^β^ (ie, a power function) have been fitted to the data using SPSS. SSBCT is the Share of SBC casualties in the Total number of road traffic casualties while BMS stands for Bicycle Modal Share. Exponent β indicates the change in SSBCT in response to changes in BMS. With β equal to 1, the growth in SSBCT with increasing BMS would be linear; β less than 1 indicates the growth in crashes would be less than linear (which would support our hypothesis). Also, the dummy variable for bicycle modal share measurement method is included as a control in these analyses.

Again, for hospitalised casualties the control variable is added to the equation to address bicycle modal share measurement method: SSBCT=α BMS^β1^ Exp(β2 Dummy). Exponent β2 indicates the effect of measurement method. It has no effect if β2 equals 0 (Exp(0)=1).

## Results

This section reports our findings on the incidence of SBC injuries (see Fatalities and Hospitalisations and ED treatments sections) and whether the proportion of SBC casualties is associated with changes in cycling's modal share (see Is bicycle modal share associated with the proportion of cycling injuries occurring in SBCs? section).

### Fatalities

Data on fatalities are derived from police recorded crash statistics. On average in European countries, 17% of cyclist fatalities are killed by SBCs, but the proportion for different countries varies between 5% and 30%.^31^ As a proportion of the total number of traffic fatalities (including car occupants, motorcyclists, etc), the share ranges between a few hundredths of a percent and 4%.^31^ ^32^ The large variation may result from relatively low number of cyclist fatalities in countries with low amounts of cycling.

### Hospitalisations and ED treatments

Tables 1 and 2 show the proportion of SBC casualties among all injured cyclists admitted to hospitals or treated at EDs. Data are derived directly from medical registrations in most studies, but some are questionnaire studies among patients. The studies vary in size: some studies based on hospital data apply to the whole population in an area. Studies based on samples tend to be smaller, focused on data provided by one or several hospitals. The sample of injured cyclists on which the proportion of SBCs is based is included as an indication of study size. The results reveal that the share of SBC victims varies between 60% and 95% of all cyclist casualties, admitted to hospital or treated at an ED. As a proportion of the total number of hospitalised traffic casualties (including car occupants, motorcyclists, etc), the share exceeds 20% in six out of 11 countries for which this proportion is known.

**Table 1:** Proportion of SBC casualties among total number of bicycle crash and road crash casualties admitted to hospital per country

Study area, study period and sources	Share of cycling in the modal split	Share (%) of SBC victims among		Number of cyclist victims on which the share is based
		Cyclists	All road crash injuries	
Netherlands (2005–2009)^4^ ^33^	31	74	41	46 100
Denmark (2011)^33^ ^36^	19	74	33	18 489
Belgium, Flanders and Brussels (2003–2007)^1^ ^33^	13	87	30	18 750
England (2011–2012)^19^ ^33^	2	80	23	16 227
Sweden (1998–2005)^5^ ^33^	17	75	23	29 788
Finland (1985–1986)*^33^ ^37^	13	65	22	127
Canada (2010–2011)^35^ ^38^	2	82	15	4126
USA, Oregon (2002–2006)^35^ ^39^ ^40^	1	67	11	414
Australia (2008–2009)^34^ ^35^	1	63	9	3811
France, Rhône county (1996–2008)^20^ ^33^	3	65	9	2578
Iran (2003)^41^	NA	62	3	420
Germany, Münster (2009–2010)*^9^ ^35^	27	72		251
New Zealand (2011)^42^ ^43^	1	78		2260

*Questionnaire study among hospitalised victims.

SBC, single-bicycle crash.

**Table 2:** Proportion of SBC casualties in the total number of cyclist crash casualties treated at emergency departments per country

Study area, study period and sources	Share (%) of SBC victims	Number of cyclist victims on which the share is based
Netherlands (2005–2009)^23^	78	6830
England, Cambridge (2003)*^44^	69	293
Germany, Göttingen (2007–2008)*^45^	60	294
Sweden (2007–2011)^18^	77	37 563
Finland (1985–1986)*^37^	79	260
Norway (2001–2002)^46^	82	991
France, Rhône county (1996–2008)^20^	71	13 684
Austria, leisure cycling (2007–2009)*^47^	80	512
USA, California, New York and North Carolina (1993–1997)^48^	63	2509
Canada (2006)^49^	89	3817
Hong Kong, Shatin, New Territories (2006)^50^	88	1315
Turkey, Central Anatolian Region (2005–2008)^51^	95	150
United Arab Emirates (2001–2003)^52^	84	200

*Questionnaire study among victims treated at an emergency department.

SBC, single-bicycle crash.

### Is bicycle modal share associated with the proportion of cycling injuries occurring in SBCs?

This section describes the relationship between the proportion of cyclist casualties (both fatalities and hospitalisations) injured in SBCs and the share of cycling in the modal split. Table 3 shows descriptive statistics and correlation coefficients. The Pearson correlation coefficients for the relationship between the share of cycling in the modal split and the share of SBC casualties among cyclist casualties are small and not significant, indicating that the variables are unrelated. The results show that the proportion of all journeys made by bicycle is not related to the proportion of cycling injuries occurring in SBCs.

**Table 3:** Descriptive statistics and the relationship between the share of SBC casualties and the share of cycling in the modal split (shares measured in percentages)

	Mean	SD	N†	Pearson correlation					
				1	2	3	4	5	6
Fatalities in European countries (2008–2010):									
1. Share of cycling in the modal split^33^	10	8	19	1	0.01	0.56*			
2. Share of SBC fatalities in the total number of cyclist fatalities^31^	17	9	20		1	0.81**			
3. Share of SBC fatalities in the total number of road traffic fatalities^31^ ^32^	2	1	18			1			
Hospitalised casualties (from table 1)									
4. Share of cycling in the modal split	11	11	12				1	0.09	0.90**
5. Share of SBC casualties in the total number of cyclist casualties	73	8	13					1	0.59
6. Share of SBC casualties in the total number of hospitalised road traffic casualties	20	12	11						1

*p<0.05; **p<0.01.

†Number of study areas for which information was available.

SBC, single-bicycle crash.

The correlation between the share of cycling in the modal split and the share of SBC casualties in the total number of road traffic casualties is significant. The higher the proportion of cycle journeys in the modal split, the higher the percentage of all road traffic injuries caused by SBCs. This is to be expected: more cyclist casualties can be expected where there are more cyclists. The high correlation also supports the validity of the bicycle modal share measures used for this study.

Linear regression analysis on the share of hospitalised SBC casualties in the total number of hospitalised casualties has been conducted and includes a control variable for bicycle modal share measurement method to check whether the measurement method affects the result. Table 4 shows the results. The correlation of 0.09 between bicycle modal share and the proportion of hospitalised SBC casualties in the total number of hospitalised cyclist casualties reduces to 0.05 after correcting for measurement method. This supports the result that measurement method did not affect the result significantly and that the proportion of all journeys made by bicycle is not related to the proportion of cycling injuries occurring in SBCs; the proportion remains constant although within a wide range of between 60% and 95%.

**Table 4:** Estimation results for regression analysis on the share of hospitalised SBC casualties in the total number of hospitalised casualties (95% Wald CI)

	Regression coefficient	Standardised regression coefficient
Constant	73.79 (63.35 to 84.24)	–
Share of cycling in the modal split	0.04 (−0.52 to 0.59)	0.05
NTS versus Gallup (dummy variable)	−1.62 (−13.28 to 10.04)	−0.11
Model fit, adjusted R^2^	2%	

NTS, National Travel Surveys; SBC, single-bicycle crash.

### How is bicycle modal share related to the proportion of all road traffic injuries that occur in SBCs?

This section explores the hypothesis that *an increase in bicycle modal share is related to a less than proportional increase in the share of SBCs among all road crash victims*. Table 5 shows the results of the analyses of the proportion of SBC casualties in the total number of road traffic casualties. In line with the hypothesis, the exponent for the growth in the share of both SBC fatalities and hospitalised SBC casualties is smaller than 1, but only the parameter for hospitalised casualties is significantly lower than 1. The latter may be due to the relatively low fatality numbers, which increases the SD. The results suggest that the higher the proportion of all journeys made by bicycle, the lower the increase in the proportion of cyclist injuries caused by SBCs will be. The coefficient for the control variable for measurement method is close to zero and has hardly any effect.

**Table 5:** Estimation results for regression analyses on the share of SBC casualties in the total number of road traffic casualties (95% Wald CI)

	Fatalities	Hospitalised casualties
Constant	0.24 (0.10 to 0.56)	10.19 (5.04 to 20.63)
Exponent for growth in bicycle modal share	0.71 (0.33 to 1.10)	0.37 (0.09 to 0.65)
NTS versus Gallup (dummy variable)	–	0.03 (−0.74 to 0.80)
Model fit, R^2^	51%	75%

NTS, National Travel Surveys; SBC, single-bicycle crash.

## Discussion

Between 60% and 95% of cyclists admitted to hospitals or treated at EDs are victims of SBCs showing the significance of this problem. As a proportion of the total number of hospitalised traffic casualties, the share averages at 20% which is about twice as high as the average bicycle modal share. The high number of cyclists injured in SBCs cause a significant health burden both directly (through the health and social care costs of injury and economic costs including absence from work and productivity losses) and indirectly (by discouraging more active and sustainable travel choices). The size of the problem warrants more research to inform and improve preventative measures. Moreover, this study shows the importance of using medical data alongside police data to achieve a more complete picture of the cycling safety issue as regards injuries admitted to hospital or treated at EDs.

In support of the hypothesis, we found that the increase in the share of SBC casualties in the total number of road crash casualties is proportionally less than the increase in the share of cycling in the modal split; increasing modal share does not appear to increase the SBC injury burden at the same rate. But this result was only statistically significant for hospitalised casualties, not for fatalities.

The proportion of cyclist casualties killed in or hospitalised due to SBCs is not found to be related to the share of cycling in the modal split. However, this probably only holds for currently observed modal shares and not for higher ones. It seems unlikely that were cycles to be used for almost 100% of journeys the injurious collisions with other cycles and pedestrians would increase sufficiently to replace the numbers of MV-B crashes, because the former are generally less serious than the latter. The authors suppose that there is a threshold in modal share of cyclists up to which increases do not result in significant increases in the proportion of cyclist casualties resulting from SBCs. Above this level, further increases in cycling as a share of all journeys will increase incidence of SBCs as a proportion of all cycle injuries. Further research into this phenomenon is required and, until then, the proven dissociation between modal share and SBC as a proportion of all injures needs to be qualified, because it may apply only to the modal share proportions of between 1% and 31% identified by and included in this report. Note that this line of reasoning is only relevant to shares of crash types and not to changes in absolute numbers of road crash casualties which is further discussed by others.^22^ ^26^

Other studies have shown that the number of cyclist casualties in collisions (per passing cyclist or per kilometre cycled) reduces as the amount of cycling increases.^22^ ^25^ ^27^ For instance, according to Jacobsen, taking into account the amount of cycling, the probability that a motorist will collide with a cyclist declines with the roughly −0.6 power of the volume of cyclists.^25^ This phenomenon—that the increase in injury is not directly proportional to the increase in the number of cyclists on the road—has been called the non-linearity of risk.^26^ The relatively constant distribution between SBCs and other crash types irrespective of the modal split suggests that the non-linearity of risk applies to all bicycle crash types including SBCs. To illustrate this, we may suppose the non-linearity only applies to MV-B crashes. In this case, the *occurrence of MV-B* crashes would decrease with increasing shares of cycling in the modal split, while the *number of SBCs* would remain constant. The result would be a decrease in the *share of MV-B* crashes and an increase in the *share of SBCs* among cycling injuries as modal share of cycling increases. Table 3 presents insignificant correlations (0.01 for fatalities; 0.09 for hospitalised casualties) showing that the latter is not the case. This result is in accordance with earlier research suggesting a non-linear relationship between risk and exposure for SBCs and suggests that this also applies to fatal SBCs.^28^

Since safety in numbers cannot offer an explanation of the non-linearity of risk of injuries that do not involve motor vehicles, what factors might explain this phenomenon? We discuss explanations that can be studied in future research. Infrastructure may be improved in response to demand from increasing numbers of cyclists, and vice versa.^27^ ^53^ For instance, good (winter) maintenance and removing or improving the visibility of obstacles can help reduce the risk of SBCs and improve conditions for cyclists, especially those with poor vision. On the contrary, in the USA where cycling enjoys a very low modal share some cities have designated sidewalks as bicycle paths, where cyclists are confronted with fixed objects such as parking metres, utility poles, signposts and trees.^54^ We cannot yet prove a causal relationship among level of cycling, quality of cycle infrastructure and incidence of SBCs, but a relationship is conceivable given the high standards regarding these aspects in countries with high amounts of cycling. A more structured comparison of infrastructure quality between countries with varying bicycle modal shares could yield valuable data to explain the non-linearity of risk and help find ways to reduce risk. We recommend investing in infrastructure that improves both cycling safety and the attractiveness of cycling, building on the growing understanding of the causes of SBCs which are presented elsewhere.^2^ ^3^ ^9^ ^10^ ^18–21^

Another line of reasoning to explain the non-linearity of risk follows from the assumption that inexperienced and cautious cyclists are at a lower risk of SBCs injury. Consistent with differences in risk aversion, female commuter cyclists and children (especially girls) prefer routes with maximum separation from motorised traffic.^55–58^ Especially these groups cycle more in countries with high amounts of cycling.^11^ ^35^ However, the elderly also run a higher risk because they are most vulnerable.^28^ It requires more research to examine whether differences in the population of cyclists between countries with varying bicycle modal shares explain part of the non-linearity of the risk of SBCs; see for example, Fyhri *et al*^59^ for groups with different risk profiles in the population of cyclists. A design as used in this study would help, but would need to be expanded with much more detailed information on crashes and bicycle usage in age groups, gender classes, income classes and so on. Instead of the modal split variable used in this study which was derived from a survey question about people's main mode of transport for daily activities, such research requires more detailed data from NTS.

Another type of research that we would like to recommend is the development and use of alternative data sources for fatally injured SBC casualties. We used police reported data for fatalities. Even though the most severe casualties are more likely to be recorded by the police, we suspect that our outcome may be an underestimate. Data from such sources are rarely reported in scientific literature, but could yield a more reliable estimate of the share of SBCs among road traffic fatalities. Regarding hospital data, we recommend research on the quality of road crash classification in medical registrations. The International Classification of Diseases is of great value for international comparisons such as this study, but it could be that reporting of certain crash types is correlated to their frequency. Data quality may be improved by restricting to a higher level of injury severity instead of hospitalisations, for example, an MAIS of 3 or more about which data may become more widely available for European countries in the future.^60^

**What is already known on the subject**

- Only a small proportion of single-bicycle crashes are recorded in police statistics, but other sources of information about their causes exist.
- More cyclists are hospitalised due to single-bicycle crashes than as a result of collisions with motorists—the reverse holds for fatalities.
- Cyclists are less likely to be involved in collisions with motor vehicles as the numbers of cyclists and amount of cycling increase.

**What this study adds**

- Between 60% and 95% of cyclists admitted to hospitals or treated at emergency departments are single-bicycle crash victims.
- The proportions of cycling fatalities and hospital admissions due to single-bicycle crashes are not found to be related to bicycle modal share.
- However, increasing bicycle modal share is associated with a reduction in the proportion of all road traffic injuries caused by single-bicycle crashes.
